# Internal medicine specialists' attitudes towards working part-time: a comparison between 1996 and 2004

**DOI:** 10.1186/1472-6963-6-126

**Published:** 2006-10-06

**Authors:** Marjolein Lugtenberg, Phil JM Heiligers, Judith D de Jong, Lammert Hingstman

**Affiliations:** 1 NIVEL – Netherlands Institute for Health Services Research, Utrecht, The Netherlands; 2 Utrecht University, Department of Social Sciences, Utrecht, The Netherlands

## Abstract

**Background:**

Although medical specialists traditionally hold negative views towards working part-time, the practice of medicine has evolved. Given the trend towards more part-time work and that there is no evidence that it compromises the quality of care, attitudes towards part-time work may have changed as well in recent years. The aim of this paper was to examine the possible changes in attitudes towards part-time work among specialists in internal medicine between 1996 and 2004. Moreover, we wanted to determine whether these attitudes were associated with individual characteristics (age, gender, investments in work) and whether attitudes of specialists within a partnership showed more resemblance than specialists' attitudes from different partnerships.

**Methods:**

Two samples were used in this study: data of a survey conducted in 1996 and in 2004. After selecting internal medicine specialists working in general hospitals in The Netherlands, the sample consisted of 219 specialists in 1996 and 363 specialists in 2004. They were sent a questionnaire, including topics on the attitudes towards part-time work.

**Results:**

Internal medicine specialists' attitudes towards working part-time became slightly more positive between 1996 and 2004. Full-time working specialists in 2004 still expressed concerns regarding the investments of part-timers in overhead tasks, the flexibility of task division, efficiency, communication and continuity of care. In 1996 gender was the only predictor of the attitude, in 2004 being a full- or a part-timer, age and the time invested in work were associated with this attitude. Furthermore, specialists' attitudes were not found to cluster much within partnerships.

**Conclusion:**

In spite of the increasing number of specialists working or preferring to work part-time, part-time practice among internal medicine specialists seems not to be fully accepted. The results indicate that the attitudes are no longer gender based, but are associated with age and work aspects such as the number of hours worked. Though there is little evidence to support them, negative ideas about the consequences of part-time work for the quality of care still exist. Policy should be aimed at removing the organisational difficulties related to part-time work and create a system in which part-time practice is fully integrated and accepted.

## Background

Traditionally, medicine was a male dominated profession where working part-time was not possible. High standards for quality and continuity of care were translated in long working hours [1,2]. In addition, the traditional ethos of medicine values single minded commitment with obligations to sacrifice personal and family needs for patient welfare and professional demands. Working part-time does not seem to fit those standards and can lead to problems regarding continuity of care, communication, and transfer of information.

However, during the last two decades the centrality of work in people's lives is perceived to be declining [1,3]. Women combine work with family roles and there is an increase in men's participation in domestic work, although gender inequity still exists [4-7]. To be able to combine both work and family duties, part-time practice has become a useful strategy in medicine [8,9]. In The Netherlands, for instance, 26% of the medical specialists worked part-time in 1996 [8] and this number has increased since then [10]. Although the prevalence of part-time workers in The Netherlands is high in comparison to other countries, increasing numbers of part-time working physicians can be found worldwide [11-13]. Moreover, since even more specialists, both male and female, are preferring to work part-time [8,10,14,15] it can be expected that part-time work will only gain more importance in the future.

As more and more specialists work or prefer to work part-time, research has focussed on the consequences of this development for the quality of care. Several studies showed that patients of part-time doctors are just as satisfied with the quality of care as patients of full-time doctors [16-20]. However, one of these studies also demonstrated that patients of physicians who worked 'overtime', i.e. more than 65 hours a week, were more satisfied with visit-based continuity of care than were patients of full-time physicians [19]. With respect to the division of time, De Jong et al. [21] showed that part-time medical specialists spend proportionally as much time on direct patient care as full-time medical specialists. In addition, students who engage in part-time training appear to be just as motivated as their full-time peers [22].

Given that part-time work is becoming more common, and that there is no evidence that part-time work compromises quality of care, one may expect that the attitudes towards working part-time among medical specialists have changed as well. In a Dutch study from 1996 it was found that the attitudes towards working part-time within a speciality were related to the number of part-time workers and that female specialists had a more positive attitude than their male counterparts [2]. Also, specialists who had flexible working times were believed to lack commitment and to be less devoted to work [23-25]. A more recent study of Evans et al. [14] demonstrated that doctors in the UK still felt that one of the perceived disadvantages of working part-time was a negative attitude from others. Similar findings were found in a recent study in Australia [22]. However, Peters et al. [26] showed that perceptions of negative attitudes from others among trainees working part-time, may not always be accurate. Although many flexible trainees experienced negative attitudes from their full-time colleagues, the majority of them appeared to have quite positive views towards flexible training.

Although some studies have focussed on the attitudes towards working part-time, little is known about the possible attitude changes and specific aspects of attitude changes in time. Moreover, research has not considered whether differences in attitudes are related to other factors such as the time invested in work or the partnership an individual participates in. Studying attitudes is important because a negative attitude towards part-time work may prevent specialists preferring to work part-time from choosing their preferred career by serving as a closure mechanism. It is known that women tend to complete specialist training in specialities where part-time work is more accepted [27]. Besides individual preferences, the constraints associated with different specialities, such as opportunities to work part-time and prevailing social norms seem to play a role [9,28]. According to Gjerberg [9] the attitudes of colleagues will have a great effect on the number of specialists who will go ahead with training as hospital specialists in part-time jobs. A more positive attitude can help part-timers achieve their ambitions and retain them within the profession. Finally, by studying attitude changes we can see which aspects have changed and which aspects are still considered to be problematic, thereby pointing to recommendations for policy.

The primary focus of this paper is therefore on the possible changes in (specific aspects of) attitudes towards working part-time of medical specialists over the years. For this purpose we selected a group of medical specialists amongst whom the attitudes towards working part-time were first measured in 1996 and again in 2004: specialists in internal medicine working in general hospitals in The Netherlands. Three specific questions will be examined:

1. To what extent and in what way did the attitude towards working part-time change between 1996 and 2004 among specialists in internal medicine?

2. What is the relationship between individual characteristics (gender, age, time invested in work) and the attitudes towards working part-time in 1996 and in 2004?

3. Are the attitudes towards working part-time of specialists in internal medicine within the same partnership more similar than attitudes of specialists in internal medicine from different partnerships?

## Methods

### Setting

Within the Dutch system of medical care most medical specialists work in a hospital setting, although they are usually not employed by the hospital. The majority are self-employed, working with other specialists of the same speciality in a partnership. A partnership can be defined as an organisational structure in which all partners are equals, mutually dependent, have a common goal, and generate their own income. Although partnerships are relatively independent entities, they can only exist within the context of the hospital. In the same way, hospitals cannot exist without medical specialists [29]. In 2000 71% of the medical specialists in The Netherlands were self-employed [29], in 2002 this figure was 75% [30]. A small proportion of medical specialists were employed by the hospital, usually working in academic hospitals.

### Study population

Data used in this article consist of two samples: data from a survey conducted in 1996 and data from a survey conducted in 2004.

#### Survey 1996

Among other groups of medical specialists, specialists in internal medicine working in general and academic hospitals in The Netherlands participated in this study. A stratified random sample was drawn from the professional register. In order to ensure that enough part-time working respondents participated, the proportion of female respondents was raised. The resulting sample consisted of 615 specialists in internal medicine. They were sent a questionnaire, including topics on the attitude towards working part-time and the time invested in work. The response was 63% (n = 390) and did not vary much with respect to gender. To be able to compare the chartacteristics of the 1996 sample to those of the 2004 sample, a weight factor for gender was composed, thereby correcting for the high number of females due to the specific method of sampling. These weighted data were used when describing the characteristics of the sample.

#### Survey 2004

Three different groups of specialists, including specialists in internal medicine, participated in this study. A questionnaire was sent to all specialists in internal medicine working in general hospitals in The Netherlands (n = 817). Among other topics, questions were asked about attitudes towards working part-time and the time invested in work. The response was 53% for internal medicine specialists (n = 411).

Since work arrangements in academic hospitals differ fundamentally from those in general hospitals, the focus of this study was solely on specialists in internal medicine working in general hospitals in The Netherlands. Therefore, 171 specialists in internal medicine of the 1996 sample and 9 specialists in internal medicine of the 2004 sample working in academic hospitals were excluded. For 39 specialists of the 2004 sample it was unknown whether they worked part-time or full-time. Consequently, they were excluded from analyses as well. After exclusion, the final sample consisted of 219 specialists in internal medicine in 1996 and 363 specialists in internal medicine in 2004.

The responding specialists in internal medicine in 1996 were compared to the population of specialists in internal medicine in 1996 with respect to gender and age. In our 1996 sample 84% of the specialists were male. For the population in 1996 this figure was 82%. With respect to age, specialists in internal medicine in our 1996 sample were slightly underrepresented in the two highest age categories (60–64 years and 65 years and older), but the differences were small. The proportion of part-time workers was not available for the population of 1996. However, the number of hours worked per week showed no significant difference between responding specialists (54.2 hours) and the population (56 hours). In our 1996 sample 56% worked in a general hospital; for the population this figure was 55%. The 1996 sample, thus, seems representative for the population in 1996. Due to a lack of information about the population of internal medicine specialists in 2004, we were not able to see whether our 2004 sample was representative for the population of internal medicine specialists working in general hospitals.

### Measures

#### Background variables

Background variables included in this study were gender and age. Age was classified into four categories: ≤ 34 years, 35–44 years, 45–54 years and ≥ 55 years. Besides these socio-demographic characteristics, the actual time invested in work (in hours), was included as a background variable. For this purpose questions were asked about the average number of hours the specialists in internal medicine worked weekly. Time invested in work was classified into three categories: < 25 hours, 25–50 hours and 50–80 hours a week.

#### Full-time or part-time

To determine whether an internal medicine specialist worked part-time or full-time, in both studies questions were asked about the formal time worked, expressed in full-time equivalents (FTE). Part-timers were defined as working less than 1,0 FTE and full-timers as working 1,0 FTE.

#### Attitudes towards working part-time

Part-time attitude was measured with 10 items using a five-point Likert-scale in 1996 (1 = I fully agree, 3 = partly agree, partly disagree and 5 = I fully disagree) and a three-point scale in 2004 (1 = I fully agree, 2 = partly agree, partly disagree and 3 = I fully disagree). The scale was devised in 1996 on the basis of 20 interviews with medical specialists in The Netherlands, concerning the (positive and negative) consequences of part-time work. The resulting scale covered different aspects of the attitude towards working part-time such as consequences of working part-time for the quality of care, consequences of working part-time for one's individual career, and consequences of working part-time for the organisation (e.g. 'If the proportion of part-time doctors increases, the flexibility of task division will be higher'). The mean score on the 10-item scale reflects an overall attitude towards part-time work. The internal consistency of the 10-item scale in both studies was high (Cronbach's alpha .77 in both studies). In order to be able to compare the 1996 data with those from 2004, the scores from 1996 were recoded into a three-point scale similar to the one used in 2004 (score 1, 2→1, score 3→2 and score 4, 5→3).

### Analyses

To answer the first research question concerning the possible changes in attitudes in time, the mean attitude towards working part-time was calculated for full-time working specialists and part-time working specialists in 1996 and 2004. Differences in attitudes between full-timers and part-timers and between 1996 and 2004 were tested using Analysis of Variance (ANOVA). To examine the relationship between individual characteristics and the attitudes towards part-time work (second research question), two more Analyses of Variance were performed: one for 1996 and one for 2004. In both analyses, working part-time was taken as a factor and gender, age and time invested as covariates. The unweighted data were used in these analyses.

The third research question, concerning the degree of resemblance in internal medicine specialists' attitudes within partnerships, was examined by calculating the Intra Class Correlation (ICC). For this purpose multilevel analyses were performed, using Mlwin. With multilevel analyses the total variation is separated in different parts: a part due to differences between medical specialists and another part due to differences between partnerships [31]. The ICC was calculated for the sample of 2004. In addition, covariates that proved to be significant from the ANOVA's were taken in account. For the specialists in internal medicine of the 1996 sample, however, no information was available as to what partnership individual specialists participated in. Therefore, it was not possible to examine the degree of resemblance in attitudes within partnerships for the sample of 1996.

## Results

### Sample characteristics: respondents in 1996 and in 2004

As can be seen in Table 1 the majority of the specialists in internal medicine in 1996 as well as in 2004 were male; however, the proportion of males was significantly higher in 1996 than in 2004 (90.9% vs. 77.7%) As expected, the proportion of part-time workers was higher in 2004 (29.2%) than in 1996 (9.1%). In addition, respondents of the 2004 sample were significantly older than respondents of the sample in 1996 (47.1 vs. 48.6). Furthermore, the two groups differed significantly with respect to mean FTE and mean number of hours worked: compared to specialists in internal medicine in 2004 the average number of FTE and hours was higher for specialists in 1996. However, for part-time working specialists the mean FTE was significantly higher in 2004 than in 1996 (0.76 vs. 0.68).

### Attitude change between 1996 and 2004

Table 2 shows the mean scores of specialists in internal medicine in 1996 and in 2004 on attitudes towards working part-time. In 1996 the mean score was 1.9, indicating a slightly negative attitude towards working part-time. Compared to part-time working specialists, full-timers had a less positive attitude. In addition, female specialists viewed part-time work more positively than did male specialists, independent of whether they were a full-timer or a part-timer.

Similar to the specialists in internal medicine in 1996, part-time working internal medicine specialists of the 2004 sample had a more positive attitude towards working part-time than did full-time working specialists. However, the mean attitude score did not differ significantly for part-time working male and female specialists and full-time working male and female specialists, indicating that gender based differences in attitude had disappeared.

Comparing the attitudes of specialists in internal medicine in 1996 to the attitudes of specialists in internal medicine in 2004, a small but significant difference is found: specialists in internal medicine in 2004 were more positive about working part-time than specialists in internal medicine in 1996. This difference can, to a great extent, be attributed to a change of attitude of part-time working male specialists in internal medicine: their score increased from 1.8 in 1996 to 2.2 in 2004. The attitude of part-time working females as well as full-time working male and female specialists in internal medicine, on the other hand, showed no significant difference between 1996 and 2004.

### Aspects of attitude (change) of full-timers and part-timers

Specific information about the attitudes and attitude changes can be found at the item level of the attitude scale. Table 3 shows the mean scores of full-time and part-time specialists in internal medicine in 1996 and in 2004 for the 10 aspects regarding the attitude towards part-time work. Specialists in internal medicine in 1996 and in 2004 were most positive about the number of part-time working specialists in their speciality: they did not think that the number of part-timers in 2004 had already reached a ceiling. In addition, specialists in internal medicine in 2004 were quite positive about the autonomy of a part-time working internist and his ability to build up a network. Full-timers viewed these aspects more positive than in 1996.

Both full-time and part-time working specialists in internal medicine in 2004 were least positive regarding the possibility of building up an independent position, when having a small part-time job at the start of a career. The scores of 1996 demonstrated the same picture. Also, full-timers in 2004 had reservations concerning investments of part-timers in overhead-tasks and the flexibility of task division with an increasing number of part-time workers. Moreover, full-time specialists in 2004 still viewed part-time work as threatening for the continuity and quality of care and expressed concerns regarding the ability of part-time specialists to maintain communication with colleagues. However, for both aspects significant changes in a positive direction were observed. Comparing part-timers to full-timers in 2004, the largest differences were found with respect to the consequences of part-time work for the continuity of care and the efficiency of part-timers: part-timers viewed these aspects far more positively than full-timers.

### Individual characteristics associated with the attitudes towards working part-time

Table 4 summarizes the association between individual characteristics and the attitude towards working part-time for specialists in internal medicine in 1996 and in 2004. Internal medicine specialists' attitudes towards working part-time in 1996 were associated with gender only, with female specialists having a more positive attitude than male specialists. Working part-time did not significantly influence the attitude towards working part-time among specialists in internal medicine in 1996, when corrected for gender. Moreover, no differences were observed with respect to age and the time invested in work.

For specialists in internal medicine in 2004 being a part-timer was significantly related to the attitude towards working part-time. Age was also associated with the attitude, with younger specialists being more positive than older ones. In addition, the number of hours worked per week significantly influenced the attitude towards working part-time, with specialists working less hours per week having a more positive attitude regarding part-time work than specialists working more hours per week. To examine whether these relations were significant for both groups, two further Analyses of Variance were performed for part-timers and full-timers in 2004 (not in Table). The results showed that only for full-time working specialists the time invested in work (β = -.12; std error = .05; p = .026) and age (β = -.11; std error = .03; p =. 001) significantly influenced the attitude towards working part-time. For part-timers, in contrast, both the time invested in work (β = -.07; std error = .10; p = .461) and age (β = -.02; std error = .05; p = .664) were not significantly related to their attitude towards part-time work.

Since the number of internal medicine specialists in both samples were quite small due to the high number of missing cases for the time invested in work, we conducted the same analyses again without taking the time invested in work into account. These analyses demonstrated the same relations as before: for the 1996 sample (n = 200) gender was the only significant predictor and for the 2004 sample (n = 326) being a part-timer and age were significantly associated with the attitude towards working part-time.

### Similarities of attitudes within partnerships

The third question we posed in this paper was whether the attitudes of specialists in internal medicine within the same partnership were more similar than attitudes of specialists in internal medicine from different partnerships. For this purpose the Intra Class Correlations (ICC) were calculated for specialists in internal medicine in 2004. As can be seen in Table 5 the Intra Class Correlation (ICC) was .04 when not correcting for other factors, indicating that 4% of the variance in attitudes can be attributed to differences between partnerships. After correcting for the three individual characteristics that turned out to be associated with this attitude (working part-time, age and the time invested in work) the ICC was .05. This means that 5% of the variance is due to differences between partnerships, when correcting for being a part-timer, age and the time invested in work. Table 5 also shows, however, that the variance in attitudes between partnerships in both analyses could not be estimated significantly. Therefore, the ICC's should only be seen as an indication of small levels of clustering and not as exact figures.

## Discussion

The present study aimed to explore to what extent and in what way internal medicine specialists' attitudes towards part-time work changed between 1996 and 2004. Moreover, we wanted to find out which individual characteristics were associated with these attitudes and whether the attitudes of specialists within a partnership showed more resemblance to each other than the attitudes of specialists from different partnerships. Overall, the results of this study illustrate that the attitude towards working part-time changed to a small extent between 1996 and 2004: specialists in internal medicine in 2004 viewed part-time work slightly more positively than specialists in internal medicine in 1996. The largest changes in a positive direction were found with respect to part-time workers' autonomy, their ability to build up a network and the consequences of part-time work for continuity of care. However, on most aspects no change or a change in negative direction was found. These findings imply that, in spite of the increasing number of part-time workers and specialists preferring to work part-time in the last decade, part-time practice among specialists in internal medicine seems not to be fully accepted.

Full-time working specialists in internal medicine in 2004 still expressed concerns about the investments of part-timers in overhead tasks and the flexibility of task division with the increasing number of doctors working part-time. However, De Jong et al. [21] showed that part-time specialists do not allocate their time differently between their tasks than full-time specialists. Moreover, although their score had increased since 1996, full-timers in 2004 still viewed part-time work as threatening for the continuity and quality of care. As mentioned before, however, there is no evidence that part-time work compromises continuity and quality of care [e.g. [17,18]]. Why negative ideas about the consequences of working part-time still exist among specialists in internal medicine needs to be further examined. On one aspect both full-timers and part-timers had strong reservations: having a small-part-time job (less than 0,3 FTE) at the start of a career. This is consistent with the study of Heiligers et al. [2] who found that the majority of medical specialists felt that part-timers had to work at least 20 to 24 hours per week.

As expected, part-time working specialists in internal medicine, both in 1996 and in 2004, had a more positive attitude towards working part-time than full-timers. With respect to gender, female specialists viewed part-time work more positively than their male counterparts. This finding is supported by several other studies [2,32]. Remarkably, we found no differences in attitudes between female and male specialists in 2004. Whereas female specialists showed about the same attitude in 1996 as in 2004, male specialists became more positive, thereby ending the gender differences in attitudes. These findings suggest that the increasing number of male specialists working or preferring to work part-time, are reflected in a more positive attitude from male specialists. The evaluation of part-time work, thus, no longer seems to be a gender based issue.

In this study the attitudes of specialists in internal medicine in 2004 were found to be associated with different factors than the attitudes of specialists in internal medicine in 1996. In 1996 gender was the only significant predictor of internal medicine specialists' attitudes towards part-time work, with females being more positive than male specialists. In 2004 gender was no longer a significant predictor. Being a part-timer, younger and working less hours a week were positively related to their attitudes in 2004. When examining full- and part-timers separately, both age and the actual time invested in work appeared to be related only to the attitudes of full-time working specialists: the more hours they work and the older they are, the less positive their attitude. This finding supports our notion that it can indeed be relevant to go beyond the division of part-timers and full-timers in two groups, by taking the actual time invested into account. It also suggests that especially specialists in internal medicine working 'over-time' have a more negative attitude towards part-time work. The finding that younger specialists in internal medicine have a more positive attitude towards part-time work is consistent with ideas about the youngest generation, the so called Generation X'ers. Among other characteristics, they are described as having a desire for flexible schedules and an emphasis on having time for family and friends [33-35]. However, a recent study of Jovic et al. [36] demonstrated that regardless of differences in perceptions about generations, the actual work hours and attitudes regarding patient care and life balance behaviours do not vary much between the generations. Moreover, it is important to realize that 'working part-time' for medical specialists being self-employed in The Netherlands still means working on average fourty hours a week [37].

Although the results of this study may suggest there have been small attitude changes with respect to part-time work among medical specialists in the last decade, one cannot extrapolate these results to other groups of medical specialists. Previous research has shown marked variation in attitudes between different medical specialities [2]. Compared to other groups of medical specialists, specialists in internal medicine in 1996 appeared to have the least positive attitude towards part-time work, aside from the orthopaedic surgeons. In 2004, however, the attitudes of specialists in internal medicine were more positive compared to these of surgeons and radiologists [37]. To be able to draw conclusions about the attitude changes of medical specialists on the whole, more studies which include several specialities are needed.

Another important finding of this study is that specialists' attitudes towards part-time work were not found to cluster much within partnerships. The low level of clustering of specialists' attitudes indicates that the attitudes towards part-time work are mostly individually based and suggests only a small effect of social influences and shared circumstances within a partnership beyond individual characteristics. This finding seems inconsistent with other studies reporting a considerable extent of similarity in attitudes within partnerships [e.g. [38,39]]. An explanation for our finding is that part-time work – though on the increase – is still relatively uncommon for specialists in internal medicine. As a result a fair number of partnerships may not have had any experience with part-time workers or specialists in internal medicine preferring to work part-time. The attitudes towards this subject may therefore predominantly reflect private opinions, since part-time work may not be an issue in these partnerships. This explanation is in line with a recent study which found that whether or not medical specialists are willing to work part-time is purely individual [40]. Presumably, the type of partnership (all full-timers, all part-timers or mixed) also plays a role with respect to the similarity of attitudes. Unfortunately however, we did not have information as to the type of partnerships specialists in internal medicine participated in.

In terms of limitations of the results, the number of part-timers was quite small for the 1996 sample. As a consequence, possibly not every association became apparent since it was more difficult to detect significant differences. Secondly, the variance in attitudes between partnerships in both analyses could not be estimated significantly. As a result we were not able to give reliable estimations as to the extent of attitude clustering within partnerships. Although the number of partnerships in our sample was not small (n = 110) and the results indicate that there is only a small extent of clustering of attitudes within partnerships, to give reliable figures a greater sample of partnerships is needed. However, since all partnerships of specialists in internal medicine in general hospitals in The Netherlands participated in this study, this was not possible. Furthermore, as there were no previously validated questionnaires about the attitude towards working part-time, a new scale was devised for the study of 1996. Although the scale had a good internal consistency, more research is needed on this subject. A further limitation of the study is that it is not longitudinal but involves two cohorts of specialists in internal medicine. In studies with a longitudinal design one can monitor the attitudes of medical specialists and the factors that influence them for several years, thereby contributing to our understanding of attitudes and attitude change.

The main strength of this study lies in the examination of the general climate for part-time work as well as the changes in the specific aspects of attitudes during the years. Moreover, it provides a picture about which individual characteristics contribute to a more positive or negative attitude. Whereas the individual characteristics that are associated with the attitudes are mainly descriptive and cannot be changed, the evaluation of the specific aspects of the attitudes can serve as useful indicators for policy. In general, the attitudes of specialists in internal medicine towards working part-time became slightly more positive over the past eight years. These findings may suggest that the time period was not long enough to produce major changes. In earlier studies it was stated that informal rules and cultural norms within the medical professions are very hard to overcome [1,24]. Male specialists in internal medicine already view part-time work as positively as female specialists and the more positive attitude of younger specialists may point towards a change in a positive direction in the future. However, since the attitude change is only apparent for some groups, e.g. female specialists show no difference in attitude over the years, getting a more positive attitude does not seem to be just a matter of time. Full-time working specialists in internal medicine still express concerns with respect to the consequences of working part-time for investments in overhead tasks, flexibility of task division, efficiency, communication and continuity of care. Policy should be aimed at creating part-time arrangements and removing the organisational difficulties related to part-time work. Moreover, it is important that all specialists, both full-timers and part-timers, have a realistic view as to the consequences of working part-time. With the increasing number of specialists opting for part-time careers it is beneficial for hospitals and other health organizations to create a system in which part-time practice is fully integrated and accepted.

## Conclusion

In spite of the increasing number of specialists seeking part-time careers, part-time practice among specialists in internal medicine seems not to be fully accepted. Gender no longer seems to be a significant predictor of the attitude towards part-time work. Rather, age and work aspects such as the number of hours worked were found to be associated with this attitude. Although there is little evidence to support them, full-time specialists in internal medicine in 2004 still expressed concerns about the consequences of part-time work for different aspects of the quality and continuity of care. Recognising and addressing these concerns is important, when integrating part-time practice within organisations. Besides removing the organisational difficulties related to part-time work, policy should be aimed at creating a realistic view as to the consequences of part-time practice, thereby contributing to more positive attitudes towards part-time work.

## Competing interests

The author(s) declare that they have no competing interests.

## Authors' contributions

ML drafted the manuscript, performed the statistical analyses, and contributed to all other aspects of the study. PH contributed to the acquisition of the data in 1996 and in 2004 and was involved in drafting the manuscript. JdJ contributed to the acquisition of the data in 2004 and critical revision of this manuscript. LH contributed to the acquisition of the data in 1996 and in 2004 and was involved in drafting the manuscript. All authors have given final approval of the submitted manuscript.

## Pre-publication history

The pre-publication history for this paper can be accessed here:



## Figures and Tables

**Table 1:** Characteristics of specialists in internal medicine working in general hospitals of the sample of 1996 and 2004

	**1996**			**2004**		
	
	Full-timers (n = 199)	Part-timers (n = 20)	Total sample (n = 219)	Full-timers (n = 257)	Part-timers (n = 106)	Total sample (n = 363)
Male (%)	188 (94.5)	11 (55)	199 (90.9)	228 (89.1)*	54 (50.5)	282 (77.7)***
Mean age (sd)	46.9 (8.2)	49.0 (9.5)	47.1 (8.4)	48.9 (7.0)**	47.8 (7.6)	48.6 (7.2)*
Mean FTE (sd)	1.0 (.00)	0.68 (.20)	.97 (.11)	1.0 (.00)	.76 (.15)*	.93 (0.14)***
Mean hours (sd)	57.1 (9.1)	38.3 (11.4)	55.9 (10.3)	53.9 (8.1)**	40.4 (14.3)	50 (11.9)***

*T*-tests were used for comparison between groups of continuous variables and chi-square test for discrete variables. All comparisons between 1996 and 2004

* p < .05; ** p < .01; *** p < .001

Note: the data of 1996 are weighted.

**Table 2:** Differences in attitudes towards working part-time between full-time and part-time workingspecialists in internal medicine and gender in 1996 and in 2004

	**1996 **(n = 200)	**2004 **(n = 327)	**Difference 1996–2004**
	
	M (sd)	M (sd)	Mean difference
*All*	1.9 (.45)	2.0 (.39)	+.13**
Full-timers	1.9 (.44)	1.9 (.37)	+.07
Part-timers	2.2 (.42)***	2.3 (.35)***	+.05
			
*Part-timers*			
Male	1.8 (.33)	2.2 (.38)	+.40**
Female	2.4 (.27)***	2.3 (.29)	-.11
			
*Full-timers*			
Male	1.8 (.42)	1.9 (.37)	+.11
Female	2.3 (.41)***	2.0 (.37)	-.25

Attitude was scored from 1–3: 1= I fully agree and 3 = I do not agree at all. A higher score reflects a more positive attitude.

*p < .05; **p < .01; ***p < .001

**Table 3:** Differences in attitudes towards part-time work between full-time and part-time working specialists in internal medicine in 1996 and in 2004

	**1996 **(n = 200)		**2004 **(n = 327)	
	
	*Part-timers*	*Full-timers*	*Part-timers*	*Full-timers*
- Part-time working is restrictive towards autonomy	2.5	2.0	2.6	2.3**
- A part-time specialist invests as much in overhead-tasks (e.g. administration) as full-time specialists	1.9	1.8	2.0	1.6**
- A small part-time job (< 0.3 FTE) at the start of a career is disastrous for building up an independent position as a specialist	1.4	1.3	1.5	1.3
- Working part-time is threatening to the continuity of patient care	2.3	1.5	2.4	1.8***
- Part-time working is negative for communication and contacts with colleagues	2.2	1.7	2.2	1.8*
- For professionalism, working part-time or full-time is not important	2.3	2.0	2.4	2.2*
- For a part-time specialist it is not possible to build up a network with other health care partners	2.5	2.0	2.6	2.3***
- The lesser hours worked the lesser efficiency will be reached	2.3	1.8	2.3	1.8
- If the proportion of part-time specialists increases, the flexibility of task division will be higher	2.1	1.8	2.0	1.7
- The number of part-time specialists in our speciality have reached a ceiling	2.9	2.6	2.7	2.5*

Attitude was scored from 1–3: 1=I fully agree and 3=I do not agree at all. A higher score reflects a more positive attitude. (Item 2, 6 and 9 were recoded).

All comparisons with *t*-tests between part-timers in 1996 and 2004 and between full-timers in 1996 and in 2004

*p < .05; **p < .01; ***p < .001

**Table 4:** Individual characteristics associated with the attitude towards working part-time

	**1996 **(n = 97)	**2004 **(n = 310)
	
	Beta (Std. error)	Beta (Std. error)
Part-time	.12 (.18)	.24 (.05)***
Gender	.55 (.14)***	.05 (.06)
Age	-.05 (.05)	-.09 (.03)**
Time invested	-.12 (.09)	-.11 (.04)*

* p < .05; **p < .01; ***p < .001

**Table 5:** Intra-Class correlations for attitudes towards working part-time and partnerships.

	Estimate (St. error)		Estimate (St. error)
Model 1		Model 2	
*Fixed*		*Fixed*	
Intercept	2.02 (.02)***	Intercept	2.02 (.02)***
*Random*		Part-time	.25 (.05)***
Var partnership	.01 (.01)	Age	-.10 (.03)***
Var specialists	.15 (.01)***	Time invested	-.11 (.04)*
		*Random*	
		Var partnership	.01 (.01)
		Var specialists	.12 (.01)***
*ICC*	*.04*	*ICC*	*.05*

(uncorrected(model 1) and corrected for significant factors (model 2) for specialists in internal medicine in 2004 (n = 319)

* p < .05; **p < .01; ***p < .001

## References

[B1] Noordenbos G, Winants Y (1994). Feiten en fricties. Sekse-asymmetrieen in zorgsystemen. [Facts and frictions Sex asymmetries in care systems].

[B2] Heiligers PhJM, Hingstman L, Marree JTC (1997). Inventarisatie deeltijdwerken onder artsen. [Review of part-time work among doctors] Utrecht: NIVEL.

[B3] Heymans R, Du Moulin M (1996). Van basisarts tot medisch specialist (M/V). From medical graduate to medical specialist (M/F).

[B4] Van der Lippe T, Siegers JJ (1994). Division of household and paid labour between partners: effects of relative wage rates and social norms. Kyklos.

[B5] Sullivan O (2000). The division of domestic labour: twenty years of change. Sociology.

[B6] Van der Lippe T, Van Dijk L (2001). Women's employment in a comparative perspective.

[B7] Milkie MA, Bianchie SM, Mattingly MJ, Robinson JP (2002). Gendered division of childrearing: ideas, realities and the relationship to parental well-being. Sex roles.

[B8] Heiligers PJM, Hingstman L (2000). Career preferences and the work-family balance in medicine: Gender differences among medical specialists. So Sci Med.

[B9] Gjerberg E (2003). Women doctors in Norway: The challenging balance between career and family Life. So Sci Med.

[B10] Heiligers PJM, de Jong JD, Hingstman L, Lugtenberg M, Groenewegen PP (2006). Integratie deeltijdwerken medisch specialisten. Verantwoording, methoden en conclusies. [Integration of part-time work among medical specialists Justification methods, and conclusions] Utrecht: NIVEL.

[B11] Visser J (2001). Negotiated flexibility in working time and labour market transitions – the case of the Netherlands.

[B12] Cooper RA, Getzen TE, McKee HJ, Laud P (2002). Economic and demographic trends signal an impending physician shortage. Health Affairs.

[B13] McMurray JE, Cohen M, Angus G, Harding J, Gavel P, Horvath J, Paice E, Schmittdiel J, Grumbach K (2002). Women in medicine: a four-nation comparison. J Am Med Women Assoc.

[B14] Evans J, Goldrace MJ, Lambert TW (2000). Views of UK medical graduates about flexible and part-time working in medicine: a qualitative study. Med Educ.

[B15] Mather HM (2001). Specialist registrars' plan for working part-time as consultants in medical specialities: questionnaire study. BMJ.

[B16] Fein OT, Garfield R (1991). Impact of physicians' part-time status on inpatients' use of medical care and their satisfaction with physicians in an academic group practice. Acad Med.

[B17] Haas JS, Cook EF, Puopolo AL, Burstin HR, Cleary PD, Brennan TA (2000). Is the professional satisfaction of general internists associated with patient satisfaction?. J Gen Intern Med.

[B18] Murray A, Safran DG, Rogers WH, Inui T, Chang H, Montgomery JE (2000). Part-time physicians: physician work load and patient-based assessments of primary care performances. Arch Fam Med.

[B19] Fairchild DG, McLoughlin KS, Gharib S, Horsky J, Portnow M, Richter J, Gagliano N, Bates DW (2001). Productivity, quality, and patient satisfaction: comparison of part-time and full-time primary care physicians. J Gen Intern Med.

[B20] Parkerton PH, Wagner EH, Smith DG, Straley HL (2003). Effect of part-time practice on patient outcomes. J Gen Intern Med.

[B21] De Jong J, Heiligers P, Groenewegen PP, Hingstman L (2006). Part-time and full-time medical specialists, are there differences in allocation of time?. BMC Health Services Research.

[B22] Postgraduate Medical Council of New South Wales (2005). Flexible Working Project, National Advisory Group & Steering Committee Meeting Report.

[B23] Lorber J, Riska E, Wegar K (1993). Why women physicians will never be true equals in the American medical profession. Gender, work and medicine Women and the medical division of labour.

[B24] Keizer M (1997). De Dokter spreekt. Professionaliteit, gender en uitsluiting in medische specialismen. [The doctor speaks Professionalism, gender and exclusion in medical specialisms] PhD Thesis.

[B25] Winants YHWM (1999). Co-assistentschappen als inwijding in de medische beroepscultuur: gender in de socialisatie tot arts. [Internship as initiation into the medical profession culture Gender in the socialisation process in medicine] PhD Thesis.

[B26] Peters E, Flett A, Challis M, Jones J (2000). Perceptions of flexible training in medicine. Hosp Med.

[B27] Gjerberg E (2002). Gender similarities in doctors' preferences – and gender differences in final specialisation. So Sci Med.

[B28] Crompton R, Harris F (1998). Gender relations and employment: the impact of occupation. Work, Employment and Society.

[B29] Groenewegen PP, Van Lindert H, Arts W, Batenburg R, Groenewegen P (2001). Vrij beroep in afhankelijkheid: de veranderende positie van medisch specialisten in de Nederlandse algemene ziekenhuizen. [Liberal profession, but dependent: the changing position of medical specialists in Dutch general hospitals]. Een kwestie van vertrouwen [A question of confidence].

[B30] Van Lindert H, Hutten H, Groenewegen PP (2003). Specialist en ziekenhuisbeleid: de klassieke organisatie verdwijnt. [Specialist and policy in hospitals: the classical organisation is disappearing] Med Contact.

[B31] Leyland AH, Groenewegen PP (2003). Multilevel modelling and public health policy. Scand J Public Health.

[B32] McMurray JE, Heiligers PJM, Shugerman RP, Douglas JA, Gangnon RE, Voss C, Costa ST, Linzer M (2005). Part-time medical practice: Where is it headed?. The American Journal of medicine.

[B33] Washburn E (2000). Are you ready for generation X?. Physician Exec.

[B34] Clausing SL, Kurtz DL, Prendeville J, Walt JL (2003). Generational diversity – the Nexters. AORN J.

[B35] Shields M, Shields M (2003). Working with Generation X Physicians. Physician Exec.

[B36] Jovic E, Wallace JE, Lemaire J (2006). The generation and gender shifts in medicine: an exploratory survey of internal medicine physicians. BMC Health Services Research.

[B37] De Jong J, Heiligers PJM, Hingstman L (2005). Tabellenboek Integratie deeltijdwerken medisch specialisten fase 1. Problemen en knelpunten bij het invoeren van deeltijdwerken. [Tables book Integration of part-time work among medical specialists Problems and bottle-necks with the realisation of part-time work] Utrecht: NIVEL.

[B38] De Jong JD, Groenewegen PP, Westert GP (2003). Mutual influences of general practitioners in partnerships. So Sci Med.

[B39] Thapar AK, Roland MO (2005). General practitioner attitudes to the care of people with epilepsy: an examination of clustering within practices and prediction of patient-rated quality of care. BMC Family Practice.

[B40] De Jong JD, Heiligers P, Groenewegen PP, Hingstman L (2005). Why are some medical specialists working part-time, while others work full-time?. Health Policy epub ahead of print.

